# Real-World Effectiveness of Fluticasone Furoate/Umeclidinium/Vilanterol Initiation in Japanese Patients with Asthma Previously on Inhaled Corticosteroid/Long-Acting β_2_-Agonist Therapy: A Retrospective Cohort Study

**DOI:** 10.3390/jcm14082566

**Published:** 2025-04-09

**Authors:** Toru Oga, Yasuhiro Gon, Masashi Takano, Risako Ito, Chifuku Mita, Isao Mukai, Stephen G. Noorduyn, Gema Requena, Masao Yarita

**Affiliations:** 1 Department of Respiratory Medicine, Kawasaki Medical School, Kurashiki 701-0192, Japan; 2 Division of Respiratory Medicine, Department of Internal Medicine, Nihon University School of Medicine, Toyko 173-8610, Japan; 3 Real World Data Analytics, Japan Development, GSK, Tokyo 107-0052, Japanmasao.2.yarita@gsk.com (M.Y.); 4 Value Evidence and Outcomes, Chief Patient Officer Organization, GSK, Tokyo 107-0052, Japan; 5 Respiratory Medical Affairs, Japan Medical Affairs, GSK, Tokyo 107-0052, Japan; 6 Real World Evidence and Health Outcomes, Global Medical, GSK, Mississauga, ON L5R 4H1, Canada; 7 Department of Health Research Methods, Evidence and Impact, McMaster University, Hamilton, ON L8S 4L8, Canada; 8 Global Value Evidence and Outcomes, Epidemiology, GSK, London WC1A 1DG, UK

**Keywords:** asthma exacerbation, adrenergic β_2_ receptor agonist, anti-asthmatic agents, corticosteroid, fluticasone furoate/umeclidinium/vilanterol, Japan, single-inhaler triple therapy

## Abstract

**Background**: Japanese guidelines recommend the addition of a long-acting muscarinic antagonist for patients with asthma uncontrolled on inhaled corticosteroid/long-acting β_2_-agonist (ICS/LABA) therapy, the effectiveness of which is evaluated here. **Methods**: Retrospective, observational, single-arm cohort study in patients with asthma initiating fluticasone furoate/umeclidinium/vilanterol (FF/UMEC/VI) following ICS/LABA, using independently analyzed data from Japanese claims databases: JMDC and Medical Data Vision (MDV). The index date was that of the first FF/UMEC/VI prescription. Outcomes were assessed during a 12-month follow-up versus a 12-month pre-index period (baseline) and included asthma exacerbations, oral corticosteroid (OCS) use, and short-acting β_2_-agonist (SABA) use. P-values associated with rate ratios (RRs) were estimated using Conditional Poisson regression. **Results**: Overall, 3229 patients in the JMDC database and 1135 in the MDV database were included. Following FF/UMEC/VI initiation, the total annualized moderate–severe asthma exacerbation rate in the JMDC database reduced from 0.50 to 0.40 per-person-per-year (PPPY) (RR [95% confidence interval]: 0.78 [0.73, 0.84]; *p* < 0.001), with similar reductions in the MDV database: 0.53 to 0.42 PPPY (0.79 [0.70, 0.89]; *p* < 0.001). In both databases, there was a 20% reduction (JMDC: 0.80 [0.73, 0.88]; *p* < 0.001; MDV: 0.80 [0.68, 0.94]; *p* = 0.005) in patients with ≥1 OCS prescription after FF/UMEC/VI initiation. The proportion of patients with ≥1 SABA canister prescription dropped by 31% 0.69 [0.57, 0.84]; *p* < 0.001) in the JMDC database and 23% (0.77 [0.66, 0.90]; *p* < 0.001) in the MDV database. **Conclusions**: This suggests FF/UMEC/VI is effective in improving asthma exacerbations and reducing OCS and SABA use in Japanese patients previously using ICS/LABA in real-world clinical practice.

## 1. Introduction

According to the Global Burden of Disease Study 2019, asthma affects approximately 260 million people worldwide [1]. In Japan, asthma is a prominent chronic respiratory disease with a prevalence of approximately 3 million [2]. The 2024 Japanese Asthma Prevention and Management Guidelines (JGL) recommend inhaled corticosteroid/long-acting β_2_-agonist (ICS/LABA) combinations as Step 2 treatment in asthma when ICS alone is insufficient and state that single-inhaler ICS/LABA treatment can result in improved adherence versus a multiple-inhaler approach [3,4]. The 2024 Practical Guidelines for Asthma Management (PGAM) recommend mid-dose ICS/LABA for adult patients with mild-to-severe asthma in Japan [5].

Despite good adherence to mid- or high-dose ICS/LABA, real-world studies in the United States (US) and Japan have shown a persisting burden of asthma symptoms in patients taking these medications and, therefore, an unmet need in asthma management [6,7]. For patients whose asthma remains uncontrolled on ICS/LABA treatment, the JGL and PGAM recommend escalating treatment based on a treatable traits approach with the addition of a long-acting muscarinic antagonist (LAMA) for patients with cough, sputum or airflow limitation, or leukotriene receptor antagonist (LTRA) for patients with rhinitis [4,5,8,9].

ICS/LAMA/LABA triple therapy has proved effective in providing additional control of asthma symptoms. For example, a network meta-analysis of five Phase III clinical studies has demonstrated the efficacy of triple over dual therapy for the treatment of uncontrolled asthma [10]. Suzuki and colleagues reported improved disease control and a reduced number of asthma exacerbations in the year after triple therapy initiation in Japanese patients who initiated multiple-inhaler triple therapy (MITT) compared with those treated with ICS/LABA [11]. Furthermore, the Phase IIIA CAPTAIN trial reported that fluticasone furoate/umeclidinium/vilanterol (FF/UMEC/VI) single-inhaler triple therapy (SITT) resulted in improved lung function versus FF/VI in patients with uncontrolled moderate or severe asthma previously on ICS/LABA [12]. Additionally, post hoc analyses of CAPTAIN have demonstrated the potential for patients to achieve clinical remission with inhaled maintenance therapies when defined as no systemic corticosteroid (SCS) use, no severe exacerbations, asthma control questionnaire (ACQ)-5 total score < 1.5, and change from baseline in trough forced expiratory volume in 1 s > 100 mL over 12 months [13,14]. Clinical remission may be used as a goal of asthma management in PGAM and JGL, defined by JGL as: (i) no oral corticosteroid (OCS) use; (ii) no exacerbations; (iii) well-controlled asthma; and (iv) optimization of lung function [3,4,5,9].

In Japan, triple therapy can be prescribed in either a multiple- or single-inhaler form; the latter being associated with higher rates of adherence and persistence [3]. Among SITT options, FF/UMEC/VI has been available for the treatment of asthma since November 2020 via the dry-powder ELLIPTA inhaler, administered in once-daily fixed doses of 100/62.5/25 or 200/62.5/25 mcg [15]. Post hoc analysis of the CAPTAIN study confirmed improved lung function, symptom control as measured by the ACQ-7, and symptoms with FF/UMEC/VI versus FF/VI specifically in Japanese patients [16]. A recent study reported FF/UMEC/VI to be well tolerated in routine clinical practice in patients with asthma in Japan [17]. Furthermore, a real-world study found that SITT was more commonly prescribed than MITT in Japanese patients, suggesting a preference for single inhalers amongst prescribing physicians [18].

Currently, there is limited real-world data demonstrating the effectiveness of adding a LAMA to ICS/LABA in terms of impact on asthma exacerbations and control. The objective of this study was to evaluate the effectiveness of FF/UMEC/VI SITT in Japanese patients with asthma who previously received ICS/LABA dual therapy.

## 2. Materials and Methods

### 2.1. Study Design

This was a retrospective, observational, single-arm cohort study analyzing data independently from two Japanese claims databases: JMDC (Toyko, Japan) and Medical Data Vision (MDV; Tokyo, Japan). The JMDC database contains administrative claims data from employment-based insurance plans for employees and their family members throughout Japan for approximately 14 million individuals [19]. The MDV database is a large administrative hospital claims database covering approximately 27% of acute care hospitals in Japan, containing data for over 45.3 million patients [20].

**Figure 1:**
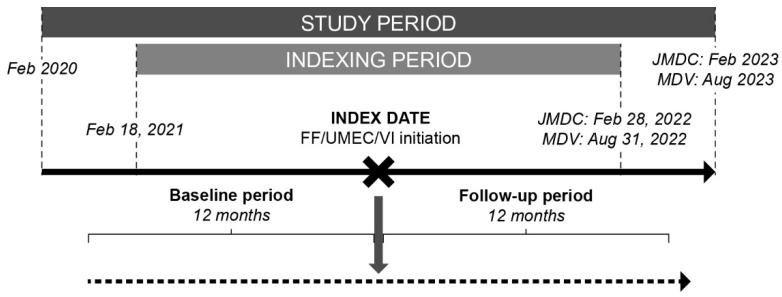
Study design. FF, fluticasone furoate; MDV, Medical Data Vision; UMEC, umeclidinium; VI, vilanterol.

The index date was defined as the date of the first FF/UMEC/VI prescription. The indexing period was from 18 February 2021 to 28 February 2022 in JMDC and 18 February 2021 to 31 August 2022 in MDV. The baseline period was defined as the 12-month period before, and excluding, the index date; and the follow-up period was the 12-month period following, and including, the index date (Figure 1).

### 2.2. Eligibility Criteria

Patients were included in the analysis if they had ≥1 prescription of FF/UMEC/VI during the indexing period, ≥1 asthma diagnosis (International Classification of Diseases, 10th Revision [ICD-10] code J45 or J46) in the same month as the index date, were ≥15 years of age at the index date, and had ≥1 prescription of single-inhaler ICS/LABA for asthma, or ≥1 overlapping day of multiple-inhaler dual therapy (concomitant use of single-inhaler ICS and single-inhaler LABA) for asthma during the baseline period. The start and end dates of the multiple-inhaled dual therapy were defined as the first and last dates with an overlap in the supply of the ICS and LABA dual therapy components. Patients were also required to have ≥12 months of continuous enrollment before and after the index date. For the JMDC database, continuous enrollment was from the first observed month and year to the last observed month and year for each patient. For the MDV database, continuous enrollment was from the first to the last medical record for each patient. Patients were excluded if they had ≥1 prescription of any SITT (including FF/UMEC/VI, mometasone furoate/glycopyrronium/indacaterol [MF/GLY/IND] or budesonide/glycopyrronium/formoterol [BUD/GLY/FOR]), ≥1 overlapping day of MITT supply during the baseline period (excluding index date), or ≥1 diagnosis of chronic obstructive pulmonary disease (ICD-10 code J41, J42, J43, or J44) prior to the index date.

### 2.3. Outcomes

Outcomes were assessed in the intent-to-treat (ITT) population throughout the 12-month follow-up period, even if patients discontinued FF/UMEC/VI treatment. Asthma exacerbations were assessed as the number of events and proportion of patients with ≥1 moderate or severe exacerbation. Moderate exacerbations were defined as an asthma-related unscheduled outpatient visit with SCS (injectable corticosteroid or OCS) prescription for 1–14 days, with no asthma-related unscheduled hospitalizations within 1 day of the outpatient visit, or another asthma-related outpatient visit with SCS for 1–14 days. Severe exacerbations were defined as an asthma-related hospitalization with an SCS prescription for 1–14 days or hospitalization within 1 day after an asthma-related unscheduled outpatient visit with an SCS prescription for 1–14 days. An unscheduled outpatient visit was defined as a visit with the code of an additional fee for an emergency department visit, after hour, late night, or holiday visit.

OCS use was measured during baseline and follow-up periods as the proportion of patients with ≥1 OCS prescription, proportion of patients with no OCS prescription, proportion of patients with reduced total OCS use from baseline to follow-up period, number of OCS prescriptions, total OCS amount prescribed, and the daily dose of OCS taken. Short-acting β_2_-agonist (SABA) use was measured as the proportion of patients with ≥1 SABA canister prescription and the number of SABA inhalations.

Exploratory outcomes included FF/UMEC/VI persistence and treatment patterns after the index. FF/UMEC/VI persistence was evaluated as the total number of days of FF/UMEC/VI use from the index date, the number of FF/UMEC/VI prescriptions, and the number of days of FF/UMEC/VI supply per prescription. Finally, the treatment patterns after the index were evaluated by documenting any controller medication prescribed during the 30 days after discontinuation of the index treatment; controller medications were not mutually exclusive.

### 2.4. Study Sample Size

The population size for the study was calculated based on the number of patients required for the sample size to be detectable with ≥80% power in the sign test to compare the proportion of patients whose total OCS decreased from the baseline to the follow-up period. This was calculated for a potential 51–80% of patients reducing their total OCS dose (Supplementary Table S1). At study start, it was estimated that 53% (*n* = 2178) of patients in the JMDC database would reduce their total OCS dose from baseline to follow-up and 59% (*n* = 240) of patients from the MDV database would have a reduction. As this was an observational study using data from medical claims databases, all patients who met the eligibility criteria were enrolled.

### 2.5. Statistical Analysis

Data from the JMDC and MDV databases were independently analyzed. Frequency and percentages were reported for categorical data; summary statistics (frequency, mean, standard deviation (SD), minimum, median, and maximum) were calculated for continuous data. For non-descriptive statistics, comparisons between baseline and follow-up periods for asthma exacerbations, patients with ≥1 OCS prescription and patients with ≥1 SABA canister prescription used rate ratio (RR), 95% confidence intervals (CIs), and *p*-values estimated from Conditional Poisson regression. RRs were calculated by dividing the number of patients with an event or the number of events by the total number of patients during the year before and after the index date. The total amount of OCS was calculated as descriptive statistics and the p-value of the sign test. The number of OCS prescriptions and OCS daily dose, as well as SABA inhalations, was calculated as descriptive statistics. Comparisons between baseline and follow-up period using the difference of outcome with 95% CIs and *p*-value of sign test. Asthma exacerbations, OCS, and SABA use were also assessed among two subgroups. Using data from the JMDC database, patient subgroups were those with body mass index (BMI) < 25 or ≥25 kg/m^2^. Using data from the MDV database, patient subgroups were those <65 or ≥65 years of age on the index date.

Sensitivity analyses were conducted where the timeframe of the baseline and follow-up periods were changed from 12 to 6 months. The baseline period was counted as 6 months before the index date to the index date. The follow-up period was counted from the day after the index date to 6 months from the index date.

## 3. Results

### 3.1. Study Population, Baseline Demographics, and Clinical Characteristics

A total of 3229 patients in the JMDC database and 1135 in the MDV database met the eligibility criteria (Supplementary Figure S1). In the JMDC database, the mean (SD) age of patients was 43.2 (12.1) years, with 3121 (96.7%) patients < 65 years; 1754 (54.3%) patients were female. In the MDV database, the mean (SD) age was 61.4 (16.2) years, with a similar number of patients < 65 years of age (*n* = 578, 50.9%) and ≥65 years (*n* = 557, 49.1%); 743 (65.5%) patients were female (Table 1). Additional baseline characteristics are shown in Supplementary Table S2.

The most common comorbidities at baseline were allergic rhinitis (74.6% and 51.3%), upper respiratory tract infection (56.1% and 26.3%) and gastroesophageal reflux disease (25.1% and 43.3% of patients) for both the JMDC and MDV databases, respectively (Supplementary Table S3).

All patients used ICS/LABA during the baseline period; concomitant controller medications were LTRA (68.5% and 59.6%) and anti-allergy medications other than LTRA (66.6% and 39.4%) for the JMDC and MDV databases, respectively (Supplementary Table S4). Similar trends in controller medication use were seen specifically in the 3 months before the index date (Supplementary Table S4).

### 3.2. Asthma Exacerbations

**Figure 2:**
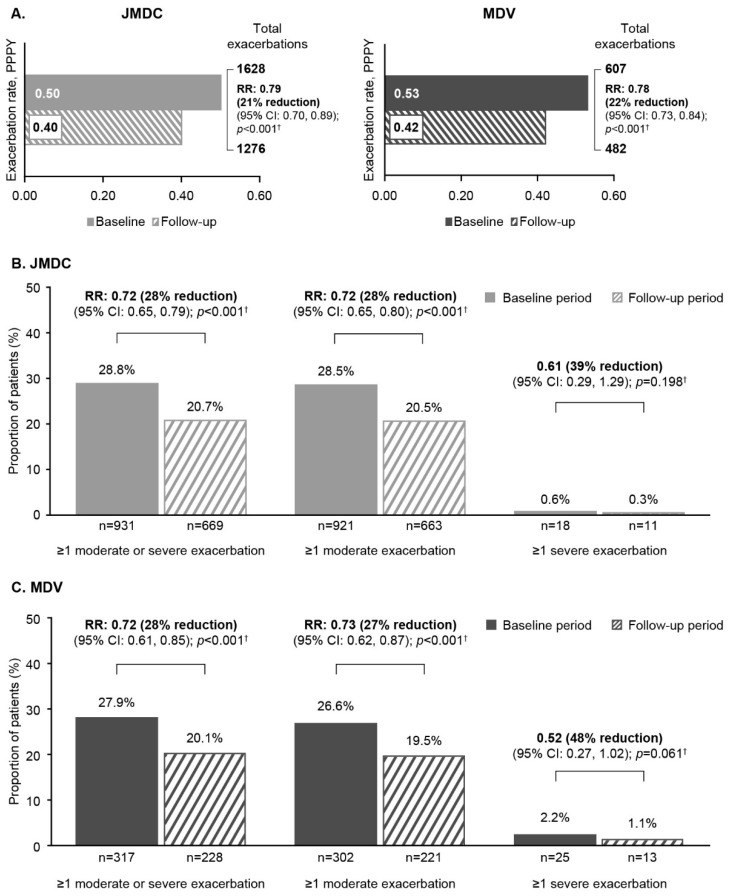
Asthma exacerbations during the baseline and follow-up periods: (**A**) total exacerbation rate in both databases; and exacerbation outcomes in (**B**) JMDC database and (**C**) MDV outcomes. ^†^ Conditional Poisson regression. CI, confidence interval; MDV, Medical Data Vision; PPPY, per-person-per-year; RR, rate ratio.

In the JMDC database, the total annualized moderate–severe exacerbation rate reduced by 22% from 0.50 to 0.40 per-person-per-year (PPPY) (RR [95% CI]: 0.78 [0.73, 0.84]; *p* < 0.001); and in MDV, the rate dropped by 21% from 0.53 to 0.42 PPPY (0.79 [0.70, 0.89]; *p* < 0.001) (Figure 2A). In the JMDC database, the proportion of patients experiencing ≥1 exacerbation was reduced by 28% for moderate (0.72 [0.65, 0.80]; *p* < 0.001) and 39% for severe exacerbations (i.e., those associated with hospitalization) (baseline: *n* = 7 [0.5%]; follow-up: *n* = 3 [0.2%]; 0.61 [0.29, 1.29]; *p* = 0.198) in the follow-up period compared with the baseline period (Figure 2B; Supplementary Table S5). There was also a 25% reduction (0.75 [0.61, 0.92]; *p* < 0.006) in unscheduled outpatient (OP) visits that resulted in SCS use in the follow-up period versus baseline. Similar results were seen in the MDV database, with reductions of 27% in the proportion of patients experiencing ≥1 moderate (0.73 [0.62, 0.87]; *p* < 0.001) and 48% in the proportion of patients experiencing ≥1 severe exacerbation (i.e., those associated with hospitalization) (baseline: *n* = 3 [0.5%]; follow-up: *n* = 6 [0.9%]; 0.52 [0.27, 1.02]; *p* = 0.055). Unscheduled OP visits resulting in SCS use were reduced by 24% (0.76 [0.44, 1.32]; *p* < 0.328) in the follow-up period versus baseline (Figure 2C; Supplementary Table S5).

Additionally, there was an 11% increase in the proportion of patients experiencing no exacerbations in the follow-up versus baseline period in both databases (JMDC: 1.11 [1.05, 1.18]; *p* < 0.001; MDV: 1.11 [1.01, 1.22]; *p* = 0.032).

### 3.3. OCS Use

**Figure 3:**
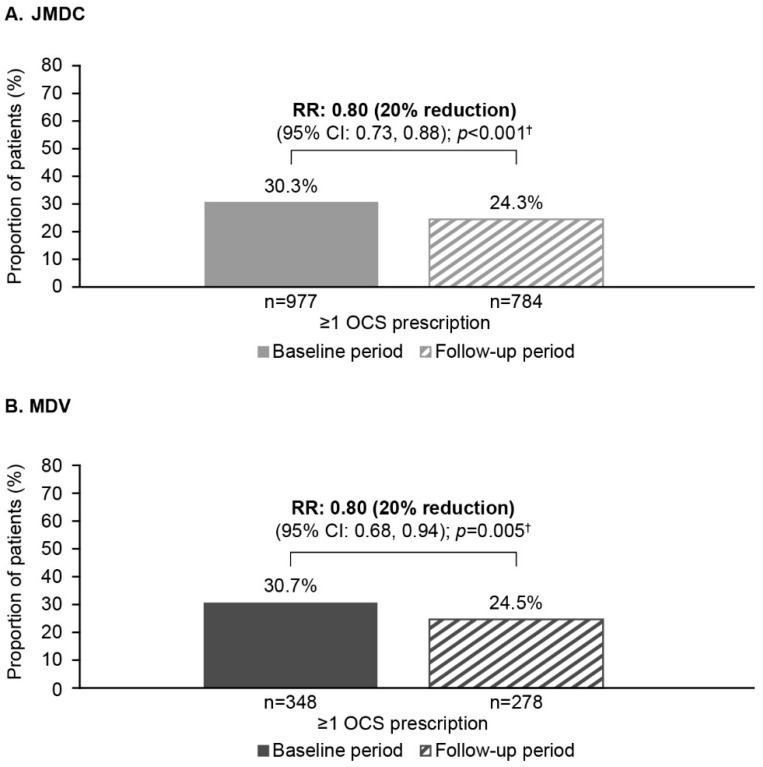
OCS prescriptions during the baseline and follow-up periods: (**A**) JMDC database and (**B**) MDV outcomes. ^†^ Conditional Poisson regression. CI, confidence interval; MDV, Medical Data Vision; OCS, oral corticosteroid; RR, rate ratio.

In both databases, there was a 20% reduction in the number of patients with ≥1 OCS prescription after FF/UMEC/VI initiation (JMDC: RR [95% CI]: 0.80 [0.73, 0.88]; *p* < 0.001; MDV: 0.80 [0.68, 0.94]; *p* = 0.005). Correspondingly, there was a 9% increase in patients with no OCS prescriptions in the follow-up versus baseline period in both databases (JMDC: 1.09 [1.03, 1.15]; *p* = 0.004; MDV: 1.09 [0.99, 1.20]; *p* = 0.084) (Figure 3).

In total, 57.7% (*n* = 725/1257) of patients with a change in total OCS use in the JMDC database had a reduction in OCS use from baseline to follow-up; this proportion was 60.8% (*n* = 253/416) in the MDV database. In the JMDC database, median (quartile [Q] 1, Q3) total OCS use among all patients was reduced from 40.1 mg (0.0, 140.0) at baseline to 0.9 mg (0.0, 105.0) in the follow-up period. A reduction from 140.0 mg (9.6, 605.9) at baseline to 103.5 mg (0.0, 600.0) in the follow-up period was reported among patients in the MDV database. Furthermore, in the JMDC database, the median (Q1, Q3) daily OCS dose decreased from 5.0 mg (0.0, 20.0) at baseline to 0.1 mg (0.0, 14.7) in the follow-up period; a reduction from 10.0 mg (0.2, 20.0) at baseline to 4.1 mg (0.0, 20.0) in the follow-up period was seen in patients in the MDV database (Table 2).

### 3.4. SABA Use

**Figure 4:**
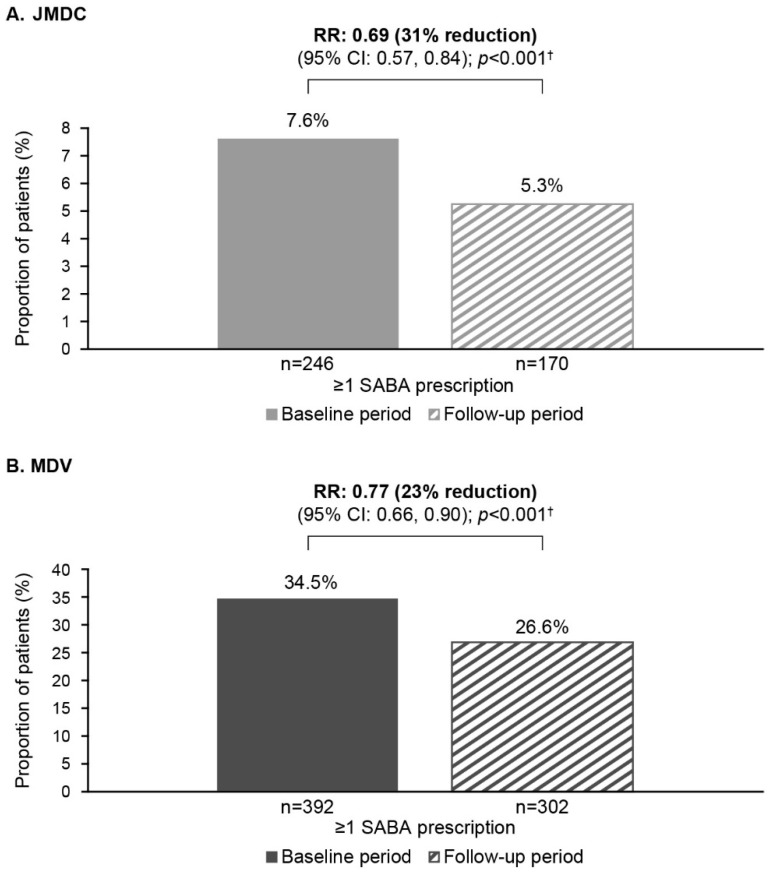
SABA use during the baseline and follow-up periods: (**A**) JMDC database and (**B**) MDV outcomes. ^†^ Conditional Poisson regression. CI, confidence interval; MDV, Medical Data Vision; RR, rate ratio; SABA, short-acting β_2_-agonist.

Use of SABA rescue medication reduced in patients in both databases following the index date. In the follow-up compared with baseline, the proportion of patients with ≥1 SABA canister prescription dropped by 31% (RR [95% CI]: 0.69 [0.57, 0.84]; *p* < 0.001) in the JMDC database and 23% (0.77 [0.66, 0.90]; *p* < 0.001) in the MDV database (Figure 4). In the JMDC database, 64.6% (*n* = 118/291) of patients with a change in SABA use had a significant reduction (*p* < 0.001) in SABA use from baseline to follow-up, while this was the case for 59.5% (*n* = 275/462) of patients in the MDV database (*p* < 0.001) (Table 2).

### 3.5. FF/UMEC/VI Persistence

During the follow-up period, mean (SD) total FF/UMEC/VI use was 137.0 (134.9) days in the JMDC database. The mean (SD) number of FF/UMEC/VI prescriptions was 4.2 (3.9), and the FF/UMEC/VI supply per prescription was 30.5 (14.9) days. In the MDV database, the mean (SD) total FF/UMEC/VI use was 260.3 (188.9) days, there were a mean (SD) number of 4.8 (3.3) FF/UMEC/VI prescriptions, and FF/UMEC/VI supply per prescription was 54.9 (26.2) days.

### 3.6. Treatment Patterns After Index

In the JMDC database, 84.0% (*n* = 2713/3229) of patients discontinued FF/UMEC/VI use in the 12 months following the index. Among these patients, in the 30 days following discontinuation, 35.2% (*n* = 954/2713) had no further visits recorded, 26.0% (*n* = 705/2713) had no controller prescribed, and 38.8% (*n* = 1053/2713) were prescribed a controller medication. Corresponding data from the MDV database showed that 59.9% (*n* = 680/1135) of patients discontinued FF/UMEC/VI use in the 12 months following the index. Within this subset, 42.9% (*n* = 292/680) had no further visits, 20.3% (*n* = 138/1135) received no controller medication, and 36.5% (*n* = 248/680) were prescribed a controller medication (Supplementary Table S6).

In both databases, the three most commonly prescribed controller medications following FF/UMEC/VI discontinuation were LTRA, ICS/LABA, and anti-allergy medications other than LTRA (Supplementary Table S6).

### 3.7. Subgroup Analyses

In the JMDC database, regardless of BMI, patients experienced fewer asthma exacerbations overall and fewer moderate exacerbations in the follow-up period compared with baseline; the number of patients experiencing ≥1 severe exacerbation at baseline was low (0.5%) (Table 3). The proportion of patients with ≥1 asthma exacerbation was lower in the follow-up versus baseline period regardless of BMI (BMI < 25 kg/m^2^: 28.2% versus 18.6%; BMI ≥ 25 kg/m^2^: 28.8% versus 22.6%). Among patients in the JMDC database with BMI < 25 kg/m^2^ and any change in total OCS use from baseline to follow-up, 59.5% (*n* = 317/533, *p* < 0.001) had a reduction in OCS use from baseline to follow-up. Among the subgroup with BMI ≥ 25 kg/m^2^, the proportion was 56.7% (*n* = 143/252, *p* = 0.037). Both subgroups showed a trend for reduction in both OCS and SABA use from baseline to follow-up, although some outcomes did not reach statistical significance (Table 3).

In the MDV database, regardless of age, patients experienced fewer asthma exacerbations overall as well as fewer moderate and severe exacerbations in the follow-up period compared with the baseline period (Table 4). The number of patients experiencing ≥1 severe exacerbation at baseline was low; 2.6% in patients under 65 years of age and 1.8% ≥ 65 years. For patients < 65 years of age, 33.9% versus 23.7% experienced ≥1 asthma exacerbation in the baseline versus follow-up; for patients ≥ 65 years of age, the trend was similar: 21.7% versus 16.3%. Among patients with a change in OCS use from baseline to follow-up, the proportion of patients with a reduction in OCS use was 62.5% (*n* = 150/240 patients, *p* < 0.001) for patients < 65 years of age and 58.5% (*n* = 103/176 patients, *p* = 0.028) for those ≥65 years of age (Table 4). Both subgroups showed a trend of reduction in OCS and SABA use although, again, some outcomes did not reach statistical significance.

### 3.8. Sensitivity Analyses

When the baseline and follow-up periods were reduced from 12 to 6 months, asthma exacerbations, OCS use, and SABA use results aligned with the main analyses for both the JMDC and MDV databases (Supplementary Table S7).

### 3.9. Discussion

In this real-world study in Japan, patients with asthma initiating treatment with FF/UMEC/VI had a significantly lower rate of annualized moderate-to-severe asthma exacerbations (JMDC: RR [95% CI]: 0.78 [0.73, 0.84]; *p* < 0.001; MDV: 0.79 [0.70, 0.89]; *p* < 0.001), and reduced OCS and rescue inhaler use in the following 12 months compared with the year before switching, during which they had ≥1 prescription of ICS/LABA. These results suggest that treatment with FF/UMEC/VI improved asthma outcomes in this patient population, which may contribute to the attainment of clinical remission.

Our study reported significant reductions in moderate (JMDC: 21%, MDV: 19%) and severe (JMDC: 42%, MDV: 43%) exacerbations in the follow-up versus baseline period, within an ITT analysis. These results build on the overall results from CAPTAIN which identified numerically fewer exacerbations in patients receiving FF/UMEC/VI compared with ICS/LABA therapy [12]. In the Japanese cohort of the CAPTAIN population, there were no clear differences in exacerbation incidence between treatment groups, likely due to the overall low incidence of exacerbation in the cohort [16]. The results from this real-world study suggest that FF/UMEC/VI is effective in reducing exacerbation frequency in Japanese patients with asthma previously on ICS/LABA.

In total, approximately 60% of patients showed significant reductions in OCS use following FF/UMEC/VI initiation. In both databases, after FF/UMEC/VI initiation, a 9% increase in the proportion of patients with no OCS prescription was observed, as well as a 20% reduction in the proportion of patients with ≥1 OCS prescription. This is of great clinical importance to patients with asthma, as there are many acute and long-term adverse effects associated with OCS use [21,22]. For example, a previous retrospective cohort study of a US medical claims database found that, when compared with no OCS prescription, the odds of experiencing an adverse event were 1.04 times higher with 1–3 OCS prescriptions in the previous year and 1.29 times higher with ≥4 OCS prescriptions [23]. This further supports the switch to FF/UMEC/VI from ICS/LABA in patients with asthma in Japan.

Similarly, there were 31% and 23% reductions in the proportion of patients with ≥1 SABA canister prescription in the year following FF/UMEC/VI initiation versus the year prior in the JMDC and MDV databases, respectively. This is in line with a real-world study in Japan that observed lower SABA use in patients 1 year after initiating FF/UMEC/VI compared with patients using ICS/LABA to manage their asthma [11]. Another recent retrospective study in the US also reported reductions in SABA use following FF/UMEC/VI initiation that were associated with decreased risk of exacerbations [24]. As increased use of SABA is associated with increased hospitalization risk, exacerbations, and mortality [25,26], lower SABA canister prescriptions associated with FF/UMEC/VI may reduce risks associated with reliever medication use.

The consistency and robustness of these results are demonstrated from the results obtained with shorter evaluation periods in the sensitivity analyses, which align with reduced exacerbation rates, OCS use, and SABA use identified in the main analyses.

The subgroup analyses by BMI and age found a trend for a reduction in the proportion of patients with exacerbations and OCS and rescue medication use following FF/UMEC/VI initiation. Since obesity is a risk factor for poor asthma control and worsening asthma severity in Japanese patients [27], the effectiveness of FF/UMEC/VI demonstrated in the subgroup of patients with high BMI is highly relevant. Furthermore, the JGL noted that elderly patients account for >90% of asthma-related deaths, and that diagnostic, therapeutic and management practices must be implemented to reduce this burden [4,8]. Therefore, reducing exacerbations and OCS use and improving asthma management in older adults are key and the effectiveness of FF/UMEC/VI in older adults is reassuring.

FF/UMEC/VI persistence was longer among patients in the MDV database versus those in the JMDC database. This difference in persistence is also seen with fewer patients discontinuing FF/UMEC/VI treatment within 12 months in the MDV database. This may be due to differences in patient characteristics and asthma severities between the two databases, particularly as the MDV database contains data from hospital settings whereas data in the JMDC database are collected mostly from general practitioner healthcare settings. Overall, there appeared to be a high proportion of patients discontinuing FF/UMEC/VI treatment across both databases within the 12-month follow-up; this is consistent with FF/UMEC/VI and triple therapy discontinuation in previous US and Japan studies [3,11]. This may partly be a result of physicians prescribing only one dose of FF/UMEC/VI. It is also possible that patients’ symptoms improved and, as such, they stopped taking the medication on their own without following the doctor’s instructions.

Our study has several strengths. This analysis included a large population of patients with asthma, using two independent claims databases, JMDC and MDV, which represent approximately 17 million and 45 million patients in Japan, respectively. Therefore, this study allowed access to a broad range of demographic and clinical data from different real-world populations. The number of patients included (*n* = 4364) is much larger than the similar preceding study in the US (*n* = 890) [24]. It must be noted, however, that the JMDC database collects health insurance society claims data, therefore representing only patients in employment and potentially underrepresenting elderly patients in Japan. Similarly, the MDV database is restricted to contracted diagnosis procedure combination hospitals, which may limit the generalizability of results to wider populations. In terms of study limitations, as the timing of ICS/LABA use during the baseline period was not specified, it is possible that patients may have used other asthma medications during the baseline period or have not used ICS/LABA immediately before the first prescription for FF/UMEC/VI. Indeed, this is suggested by data focusing on controller medications taken in the 3 months before the index. It is also possible that the OCS prescriptions recorded may not have been specifically for the treatment of asthma or may have been prescribed as low-dose maintenance therapy. Additionally, this study spanned the COVID-19 pandemic; results must be interpreted carefully to take into consideration the influence of the pandemic on the number of hospitalizations and outpatient visits. Another limitation to note is that the data in this analysis were derived from hospital or pharmacy claims, which record medications prescribed but are unable to clarify whether the medication was used, and if so, whether it was used as prescribed. Another limitation is the lack of a suitable biomarker to support the decision to prescribe triple therapy; the nature of this study did not allow for more personalized treatment strategies and phenotype identification. Finally, diagnosis (ICD-10) codes were only captured at the month level, meaning that an exact date was not recorded. Therefore, it was not possible to explicitly link diagnoses to prescriptions, and instead, a diagnosis observed within the same calendar month was often used as a proxy measure. This may lead to potential overestimation or underestimation of the asthma conditions related to the index prescriptions.

Overall, these results provide further evidence for the benefits of escalating to FF/UMEC/VI from ICS/LABA dual therapy, aiding treatment decisions in real-life clinical practice in Japan. While current Japanese asthma guidelines recommend escalation to ICS/LAMA/LABA if symptoms persist with ICS/LABA treatment [8,9], earlier initiation of triple therapy in the treatment paradigm may contribute to patients meeting components of clinical remission and achieving long-term treatment goals.

## 4. Conclusions

In conclusion, to our knowledge, this is one of the first studies to report real-world evidence showing the impact of FF/UMEC/VI SITT for patients with asthma in routine clinical practice in Japan and is the first study to report asthma exacerbation data in this patient population. This real-world study found a statistically significant reduction in exacerbations as well as a reduction in the use of OCS and SABA in Japanese patients who escalated to FF/UMEC/VI from ICS/LABA. These benefits associated with escalating FF/UMEC/VI from ICS/LABA will contribute to the possible attainment of clinical remission defined by PGAM [9] and JGL [8], at least in terms of no OCS use, no exacerbations, and well-controlled asthma.

## Figures and Tables

**Table 1:** Baseline demographics and clinical characteristics.

Patient Characteristics	JMDC Database (*N* = 3229)	MDV Database (*N* = 1135)
**Age on index date, years**		
Mean (SD)	43.2 (12.1)	61.4 (16.2)
Median (Q1, Q3)	43.0 (35.0, 52.0)	64.0 (50.0, 74.0)
**Age groups, *n* (%)**		
<65 years	3121 (96.7)	578 (50.9)
≥65 years	108 (3.3)	557 (49.1)
**Sex, n (%)**		
Female	1754 (54.3)	743 (65.5)
**Duration of medical record, months**		
Mean (SD)	64.5 (36.8)	65.4 (35.1)
Median (Q1, Q3)	57.0 (36.0, 80.0)	61.0 (36.0, 90.0)
**Duration of asthma, months**		
Mean (SD)	40.4 (34.2)	44.9 (35.6)
Median (Q1, Q3)	33.0 (14.0, 57.0)	36.0 (17.0, 70.0)
**BMI, kg/m^2^, *n* (%)**	2076 (64.3)	204 (18.0)
Mean (SD)	23.8 (4.6)	25.2 (5.8)
Median (Q1, Q3)	23.1 (20.6, 26.0)	24.7 (21.1, 28.5)
**Habitually smoke or smoking history, *n* (%)**		
Yes	304 (9.4)	140 (12.3)
No	1703 (52.7)	55 (4.8)
Missing	1222 (37.8)	940 (82.8)
**Lung function test, *n* (%)**		
Receipt for lung function test identified	1507 (46.7)	528 (46.5)
**FF/UMEC/VI dose at index date, *n* (%)**		
FF/UMEC/VI 100/62.5/25 μg	1109 (34.3)	440 (38.8)
FF/UMEC/VI 200/62.5/25 μg	2120 (65.7)	695 (61.2)

BMI, body mass index; FF, fluticasone furoate; MDV, Medical Data Vision; Q1/3, quartile 1/3; SD, standard deviation; UMEC, umeclidinium; VI, vilanterol.

**Table 2:** Total OCS supply and SABA use during the baseline and follow-up periods.

	JMDC Database (*N* = 3227)		MDV Database (*N* = 1134)	
	Baseline	Follow-Up	Baseline	Follow-Up
**Patients with reduction in OCS use in population with a change in OCS use from baseline to follow-up**				
*n*/*N* (%); ^†^ *p*-value	725/1257 (57.7); *p* < 0.001		253/416 (60.8); *p* < 0.001	
**Patients with ≥1 OCS prescription**				
Patients with OCS prescription in baseline or follow-up period, *n* (%)	1304 (40.4)		426 (37.6)	
Number of OCS prescriptions, median (Q1, Q3)	1.0 (0.0, 2.0)	1.0 (0.0, 2.0)	2.0 (1.0, 5.0)	1.0 (0.0, 5.0)
Total amount of OCS, median mg (Q1, Q3)	40.1 (0.0, 140.0)	0.9 (0.0, 105.0)	140.0 (9.6, 605.9)	103.5 (0.0, 600.0)
Daily dose of OCS, median mg (Q1, Q3)	5.0 (0.0, 20.0)	0.1 (0.0, 14.7)	10.0 (0.2, 20.0)	4.1 (0.0, 20.0)
**Patients with a reduction in SABA use in the population with a change in SABA use from baseline to follow-up**				
*n*/*N* (%); ^†^ *p*-value	188/291 (64.6); *p* < 0.001		275/462 (59.5); *p* < 0.001	
**Descriptive statistics among patients who are prescribed SABA**				
Patients with SABA prescription in baseline or follow-up periods, *n* (%)	328 (10.2)		506 (44.6)	
Number of SABA inhalations, median (Q1, Q3)	200 (2.0, 200.0)	115 (0.0, 200.0)	100 (100.0, 200.0)	100 (0.0, 200.0)

Data exclude those with OCS outliers. ^†^ Calculated as: (Number of patients with reduction)/(Number of patients with reduction or increase) × 100. MDV, Medical Data Vision; OCS, oral corticosteroid; Q1/3, quartile 1/3; SABA, short-acting β_2_-agonist.

**Table 3:** BMI subgroup analyses (JMDC database).

	BMI < 25 (*n* = 1416)		BMI ≥ 25 (*n* = 658)	
	Baseline	Follow-Up	Baseline	Follow-Up
**Patients with ≥1 moderate-to-severe asthma exacerbation**				
*n* (%)	399 (28.2)	264 (18.6)	190 (28.8)	149 (22.6)
RR (95% CI); *p*-value	0.66 (0.57, 0.77); *p* < 0.001		0.78 (0.63, 0.97); *p* = 0.026	
**Patients with ≥1 moderate exacerbation**				
*n* (%)	395 (27.9)	263 (18.6)	187 (28.4)	145 (22.0)
RR (95% CI); *p*-value	0.67 (0.57, 0.78); *p* < 0.001		0.78 (0.62, 0.96); *p* = 0.021	
**Patients with ≥1 severe exacerbation**				
*n* (%)	7 (0.5)	3 (0.2)	3 (0.5)	6 (0.9)
RR (95% CI); *p*-value	0.43 (0.11, 1.66); *p* = 0.219		2.00 (0.50, 8.00); *p* = 0.326	
**Patients with ≥1 OCS prescription**				
*n* (%)	411 (29.0)	319 (22.5)	201 (30.5)	159 (24.2)
RR (95% CI); *p*-value	0.78 (0.67, 0.90); *p* < 0.001		0.79 (0.64, 0.97); *p* = 0.027	
**Patients with no OCS prescriptions**				
*n* (%)	1005 (71.0)	1097 (77.5)	457 (69.5)	499 (75.8)
RR (95% CI); *p*-value	1.09 (1.00, 1.19); *p* = 0.044		1.09 (0.96, 1.24); *p* = 0.174	
**Patients with a reduction in OCS use in the population with a change in OCS use from baseline to follow-up**				
*n*/*N* (%); ^†^ *p*-value	317/533 (59.5); *p* < 0.001		143/252 (56.7); *p* = 0.037	
**Patients with ≥1 SABA canister prescription**				
*n* (%)	99 (7.0)	64 (4.5)	46 (7.0)	39 (5.9)
RR (95% CI); *p*-value	0.65 (0.47, 0.89); *p* = 0.006		0.85 (0.55, 1.30); *p* = 0.448	
**Patients with ≥1 OCS prescription**				
Patients with ≥1 OCS prescription in baseline or follow-up periods, *n*/*N* (%) ^†^	551/1416 (38.9)		263/658 (40.0)	
Number of OCS prescriptions, median (Q1, Q3)	1.0 (0.0, 2.0)	1.0 (0.0, 2.0)	1.0 (1.0, 3.0)	1.0 (0.0, 2.0)
Total amount of OCS, median mg (Q1, Q3)	30.1 (0.0, 140.0)	0.6 (0.0, 100.0)	60.0 (0.2, 169.2)	3.0 (0.0, 145.0)
Daily dose of OCS, median mg (Q1, Q3)	5.0 (0.0, 20.0)	0.1 (0.0, 12.5)	6.2 (0.0, 20.0)	0.1 (0.0, 20.0)
Number of SABA inhalations, median (Q1, Q3)	200.0 (7.0, 200.0)	0.0 (0.0, 200.0)	200.0 (0.0, 200.0)	200.0 (0.0, 400.0)

Data exclude those with OCS outliers. ^†^ Calculated as: (Number of patients with reduction)/(Number of patients with reduction or increase) × 100. CI, confidence interval; OCS, oral corticosteroid; Q1/3, quartile 1/3; RR, rate ratio; SABA, short-acting β_2_-agonist.

**Table 4:** Age subgroup analyses (MDV database).

	Age < 65 years (*n* = 578)		Age ≥ 65 years (*n* = 556)	
	Baseline	Follow-Up	Baseline	Follow-Up
**Patients with ≥1 moderate-severe asthma exacerbation**				
*n* (%)	196 (33.9)	137 (23.7)	121 (21.7)	91 (16.3)
RR (95% CI); *p*-value	0.70 (0.56, 0.87); *p* = 0.001		0.75 (0.57, 0.99); *p* = 0.040	
**Patients with ≥1 moderate exacerbation**				
*n* (%)	187 (32.4)	131 (22.7)	115 (20.6)	90 (16.2)
RR (95% CI); *p*-value	0.70 (0.56, 0.88); *p* = 0.001		0.78 (0.59, 1.03); *p* = 0.081	
**Patients with ≥1 severe exacerbation**				
*n* (%)	15 (2.6)	8 (1.4)	10 (1.8)	5 (0.9)
RR (95% CI); *p*-value	0.53 (0.23, 1.26); *p* = 0.151		0.50 (0.17, 1.46); *p* = 0.205	
**Patients with ≥1 OCS prescription**				
*n* (%)	204 (35.3)	162 (28.0)	144 (25.9)	116 (20.9)
RR (95% CI); *p*-value	0.79 (0.65, 0.98); *p* = 0.028		0.81 (0.63, 1.03); *p* = 0.083	
**Patients with no OCS prescriptions**				
*n* (%)	374 (64.7)	416 (72.0)	412 (74.1)	440 (79.1)
RR (95% CI); *p*-value	1.11 (0.97, 1.28); *p* = 0.135		1.07 (0.93, 1.22); *p* = 0.337	
**Patients with a reduction in OCS use in the population with a change in OCS use from baseline to follow-up**				
*n* (%); ^†^ *p*-value	150/240 (62.5); *p* < 0.001		103/176 (58.5); *p* = 0.028	
**Patients with ≥1 SABA canister prescription**				
*n* (%)	243 (42.0)	177 (30.6)	149 (26.8)	125 (22.4)
RR (95% CI); *p*-value	0.73 (0.60, 0.88); *p* = 0.001		0.84 (0.66, 1.06); *p* = 0.147	
**Patients with ≥1 OCS prescription**				
Patients with ≥1 OCS prescription in baseline or follow-up periods; *n* (%) ^†^	248/578 (42.9)		178/556 (32.0)	
Number of OCS prescriptions, median (Q1, Q3)	2.0 (1.0, 4.0)	1.0 (0.0, 4.0)	2.0 (1.0, 6.0)	1.0 (0.0, 6.0)
Total amount of OCS, median mg (Q1, Q3)	150.0 (11.2, 585.0)	120.0 (0.0, 495.0)	120.0 (5.0, 662.5)	78.4 (0.0, 750.0)
Daily dose of OCS, median mg (Q1, Q3)	15.4 (0.7, 25.0)	5.3 (0.0, 20.0)	7.6 (0.1, 20.0)	2.2 (0.0, 12.5)
Number of SABA inhalations, median (Q1, Q3)	100.0 (100.0, 200.0)	100.0 (0.0, 200.0)	100.0 (0.0, 200.0)	100.0 (0.0, 200.0)

Data exclude those with OCS outliers. ^†^ Calculated as: (Number of patients with reduction)/(Number of patients with reduction or increase) × 100. CI, confidence interval; MDV, Medical Data Vision; OCS, oral corticosteroid; Q1/3, quartile 1/3; RR, rate ratio; SABA, short-acting β_2_-agonist.

## Data Availability

Data used for this study were obtained from the JMDC and MDV databases. For inquiries regarding the database analyzed in this study, please contact JMDC (https://www.jmdc.co.jp/en/) or MDV (https://en.mdv.co.jp/).

## Supplementary Materials

The following supporting information can be downloaded at: https://www.mdpi.com/article/10.3390/jcm14082566/s1, Supplementary Table S1: Minimum sample size required for population of patients reducing total OCS dose from baseline to follow-up period to be detected with ≥80% power; Supplementary Table S2: Additional patient baseline characteristics; Supplementary Table S3: Baseline comorbidities of interest; Supplementary Table S4: Controller medication use during the baseline period; Supplementary Table S5: Asthma exacerbations during the baseline and follow-up periods; Supplementary Table S6: Treatment patterns after index; Supplementary Table S7: Sensitivity analysis; Supplementary Figure S1: Patient disposition.

## Abbreviations

The following abbreviations are used in this manuscript: ACQ, Asthma Control Questionnaire; BMI, body mass index; BUD, budesonide; CI, confidence interval; COPD, chronic obstructive pulmonary disease; COVID-19, Coronavirus Disease 2019; FF, fluticasone furoate; FOR, formoterol; GLY, glycopyrronium; ICS, inhaled corticosteroid; IND, indacaterol; ITT, intent-to-treat; JGL, Japanese Asthma Prevention and Management Guidelines; LABA, long-acting β_2_-agonist; LAMA, long-acting muscarinic antagonist; LTRA, leukotriene receptor antagonist; MDV, Medical Data Vision; MF, mometasone furoate; MITT, multiple-inhaler triple therapy; OCS, oral corticosteroid; OP, outpatient; PGAM, Practical Guidelines for Asthma Management; PPPY, per-person-per-year; Q1/3, quartile 1/3; RR, rate ratio; SABA, short-acting β_2_-agonist; SCS, systemic corticosteroid; SD, standard deviation; SITT, single-inhaler triple therapy; UMEC, umeclidinium; US, United States; VI, vilanterol.
