# Supplementary material for: Real-World Effectiveness of Fluticasone Furoate/Umeclidinium/Vilanterol Initiation in Japanese Patients with Asthma Previously on Inhaled Corticosteroid/Long-Acting β2-Agonist Therapy: A Retrospective Cohort Study
Source: J Clin Med. 2025 Apr 9;14(8):2566. doi: 10.3390/jcm14082566 (PMC12028120; doi:10.3390/jcm14082566)
Supplement: Supplementary file 1 [file jcm-14-02566-s001.zip › jcm-3517349-supplementary.pdf]

Supplementary materials

Supplementary Table S1: Minimum sample size required for population of patients reducing total OCS supply from baseline to follow-up period to be detected with ≥80% power

Proportion of patients with reduction in total amount of OCS supply, %	Number of patients
0.51	19620
0.52	4904
0.53	2178
0.54	1225
0.55	783
0.56	543
0.57	399
0.58	305
0.59	240
0.60	194
0.61	160
0.62	134
0.63	114
0.64	98
0.65	85
0.66	75
0.67	66
0.68	59
0.69	52
0.70	47
0.71	43
0.72	39
0.73	35
0.74	32
0.75	29
0.76	27
0.77	25
0.78	23
0.79	21
0.80	20

OCS, oral corticosteroid.

Supplementary Table S2: Additional patient baseline characteristics.

Patients, n (%)	JMDC database (N=3229)	MDV database (N=1135)
**Medical facility scale†**		
Hospital (≥100 beds)	215 (6.7)	-
GP (≤99 beds)	3014 (93.3)	-
**Management body†**		
Public	25 (0.8)	-
University	38 (1.2)	-
Other hospital	188 (5.8)	-
Clinic	2978 (92.2)	-
**Hospital scale‡**		
<200 beds	-	155 (13.7)
200–499 beds	-	636 (56.0)
≥500 beds	-	344 (30.3)
**Year and quarter of index date**		
2021, Feb–Mar	355 (11.0)	69 (6.1)
2021, Apr–Jun	811 (25.1)	222 (19.6)
2021, Jul–Sep	676 (20.9)	201 (17.7)
2021, Oct–Dec	958 (29.7)	234 (20.6)
2022, Jan–Mar	429 (13.3)	192 (16.9)
2022, Apr–Jun†	0 (0.0)	173 (15.2)
2022, Jul†	0 (0.0)	44 (3.9)

†Output in case JMDC; ‡output in case MDV.

GP, general practitioner; MDV, Medical Data Vision.

Supplementary Table S3: Baseline comorbidities of interest.[†]

Patients, n (%)	JMDC database (N=3229)	MDV database (N=1135)
Allergic rhinitis	2410 (74.6)	582 (51.3)
Upper respiratory tract infection[‡]	1810 (56.1)	298 (26.3)
Gastroesophageal reflux disease	809 (25.1)	492 (43.3)
Sinusitis	804 (24.9)	165 (14.5)
Depressive disorders	264 (8.2)	66 (5.8)
Anxiety disorders	206 (6.4)	57 (5.0)
Pneumonia	164 (5.1)	114 (10.0)
COVID-19	102 (3.2)	0 (0.0)
Obstructive sleep apnea	82 (2.5)	34 (3.0)
Acute respiratory failure	35 (1.1)	33 (2.9)
Eosinophilic disorders[§]	13 (0.4)	24 (2.1)

[†]Comorbidities were not mutually exclusive; [‡]does not include sinusitis; [§]includes eosinophilic pneumonia, eosinophilic bronchitis, acute eosinophilic pneumonia, chronic eosinophilic pneumonia, tropical pulmonary eosinophilia, simple pulmonary eosinophilia and allergic pneumonia.

COVID-19, Coronavirus Disease 2019; MDV, Medical Data Vision.

Supplementary Table S4: Controller medication use during the baseline period.

Patients	JMDC database (N=3229)	MDV database (N=1135)	JMDC database (N=3229)	MDV database (N=1135)
	12-month baseline period		3 months before index date	
**ICS, n (%)**				
Any	174 (5.4)	50 (4.4)	71 (2.2)	16 (1.4)
Fluticasone furoate	74 (2.3)	16 (1.4)	30 (0.9)	6 (0.5)
Fluticasone propionate	28 (0.9)	9 (0.8)	6 (0.2)	3 (0.3)
Budesonide	26 (0.8)	13 (1.1)	11 (0.3)	2 (0.2)
Budesonide inhalation suspension	18 (0.6)	2 (0.2)	11 (0.3)	0 (0.0)
Ciclesonide	11 (0.3)	10 (0.9)	6 (0.2)	4 (0.4)
Beclomethasone dipropionate	11 (0.3)	2 (0.2)	4 (0.1)	1 (0.1)
Mometasone furoate	13 (0.4)	0 (0.0)	6 (0.2)	0 (0.0)
**LABA (inhalant)**				
n (%)	8 (0.2)	0 (0.0)	3 (0.1)	0 (0.0)
**LAMA (inhalant)**				
n (%)	3 (0.1)	4 (0.4)	2 (0.1)	4 (0.4)
**ICS/LABA (inhalant)**				
Any	3229 (100.0)	1135 (100.0)	2614 (81.0)	935 (82.4)
Fluticasone furoate	1952 (60.5)	777 (68.5)	1554 (48.1)	644 (56.7)
Fluticasone propionate	766 (23.7)	210 (18.5)	548 (17.0)	137 (12.1)
Budesonide	868 (26.9)	261 (23.0)	635 (19.7)	185 (16.3)

Budesonide inhalation suspension	0 (0.0)	0 (0.0)	0 (0.0)	0 (0.0)
Ciclesonide	2 (0.1)	0 (0.0)	1 (0.0)	0 (0.0)
Beclomethasone dipropionate	3 (0.1)	0 (0.0)	2 (0.1)	0 (0.0)
Mometasone furoate	7 (0.2)	0 (0.0)	7 (0.2)	0 (0.0)
**LTRA**				
n (%)	2211 (68.5)	676 (59.6)	1741 (53.9)	555 (48.9)
**Theophyllines**				
n (%)	477 (14.8)	170 (15.0)	330 (10.2)	127 (11.2)
**Biologics**[†]				
n (%)	17 (0.5)	46 (4.1)	12 (0.4)	36 (3.2)
**Transdermal LABA**				
n (%)	314 (9.7)	49 (4.3)	162 (5.0)	25 (2.2)
**Anti-allergy medications other than LTRA**				
n (%)	2150 (66.6)	447 (39.4)	1480 (45.8)	326 (28.7)

[†]Includes benralizumab, dupilumab, mepolizumab, omalizumab and tezepelumab.

ICS, inhaled corticosteroid; LABA, long-acting β_2-agonist; LAMA, long-acting muscarinic antagonist; LTRA, leukotriene receptor antagonists; MDV, Medical Data Vision.

Supplementary Table S5: Asthma exacerbations during the baseline and follow-up periods.

	JMDC (N=3229)		MDV (N=1135)	
	**Baseline**	**Follow-up**	**Baseline**	**Follow-up**
**Moderate exacerbations**				
**Unscheduled OP visit with SCS**†				
Patients, n (%)	210 (6.5)	158 (4.9)	29 (2.6)	22 (1.9)
RR (95% CI); *p-value*‡	0.75 (0.61, 0.92); p=0.006		0.76 (0.44, 1.32); p=0.328	
**Other OP visit with SCS**				
Patients, n (%)	741 (22.9)	529 (16.4)	279 (24.6)	209 (18.4)
RR (95% CI); *p-value*‡	0.71 (0.64, 0.80); p<0.001		0.75 (0.63, 0.90); p=0.001	
**Severe exacerbations**				
**Hospitalization with SCS**				
Patients, n (%)	11 (0.3)	4 (0.1)	23 (2.0)	9 (0.8)
RR (95% CI); *p-value*‡	0.36 (0.12, 1.14); p=0.083		0.39 (0.18, 0.85); p=0.017	
**Unscheduled OP visit leading to hospitalization with SCS**§				
Patients, n (%)	8 (0.2)	7 (0.2)	2 (0.2)	4 (0.4)
RR (95% CI); *p-value*‡	0.88 (0.32, 2.41); p=0.796		2.00 (0.37, 10.92); p=0.423	

†An unscheduled OP visit was defined as a visit with the code of additional fee for emergency department visit, after hour, late night, or holiday visit; ‡Conditional Poisson regression; §an asthma-related hospitalization with SCS prescription for 1–14 days or a hospitalization within 1 day after an unscheduled asthma-related OP visit with SCS prescription for 1–14 days.

CI, confidence interval; MDV, Medical Data Vision; OP, outpatient; SCS, systemic corticosteroid; RR, rate ratio.

Supplementary Table S6: Treatment patterns after index.

**Patients**	**JMDC database (N=3229)**	**MDV database (N=1135)**
**Continued ICS/LABA/LAMA treatment up to 12 months**		
n (%)	516 (16.0)	455 (40.1)
**Discontinued treatment prior to 12 months, n (%)**		
Population	2713 (84.0)	680 (59.9)
Continuous enrollment ended	1 (0.0)	2 (0.3)
Had no visit	954 (35.2)	292 (42.9)
Had no controller medication of interest	705 (26.0)	138 (20.3)
Had controller medication of interest,	1053 (38.8)	248 (36.5)
LTRA,	578 (54.9)	125 (50.4)
ICS/LABA (inhalant)	577 (54.8)	140 (56.5)
Anti-allergy medications other than LTRA	550 (52.2)	74 (29.8)
Theophyllines	107 (10.2)	31 (12.5)
IND/GLY/MF	90 (8.5)	25 (10.1)
ICS	32 (3.0)	6 (2.4)
Transdermal LABA	23 (2.2)	5 (2.0)
MITT	21 (2.0)	10 (4.0)
LAMA (inhalant)	8 (0.8)	6 (2.4)
Biologics[†]	7 (0.7)	22 (8.9)
LABA (inhalant)	1 (0.1)	0 (0.0)
FF/UMEC/VI	0 (0.0)	0 (0.0)

[†]Includes benralizumab, dupilumab, mepolizumab, omalizumab and tezepelumab.

FF, fluticasone furoate; GLY, glycopyrronium; ICS, inhaled corticosteroid; IND, indacaterol; LABA, long-acting β_2-agonist; LAMA, long-acting muscarinic antagonist; LTRA, leukotriene receptor antagonists; MDV, Medical Data Vision; MF, mometasone furoate; MITT, multiple-inhaler triple therapy; UMEC, umeclidinium; VI, vilanterol.

Supplementary Table S7: Sensitivity analysis.

	**JMDC (N=3229)**		**MDV (N=1135)**	
	**Baseline**	**Follow-up**	**Baseline**	**Follow-up**
**Patients with ≥1 moderate or severe asthma exacerbation**				
n (%)	777 (24.1)	441 (13.7)	244 (21.5)	164 (14.4)
RR (95% CI); *p-value*†	0.57 (0.50, 0.64); *p*<0.001		0.67 (0.55, 0.82); *p*<0.001	
**Total asthma exacerbations**				
Exacerbations, n‡ (Rate, PPPY)	1099 (0.68)	663 (0.41)	369 (0.65)	271 (0.48)
RR (95% CI); *p-value*†	0.60 (0.55, 0.66); *p*<0.001		0.73 (0.63, 0.86); *p*<0.001	
**Patients with ≥1 OCS prescription**				
n (%)	806 (25.0)	517 (16.0)	288 (25.4)	224 (19.7)
RR (95% CI); *p-value*†	0.64 (0.57, 0.72); *p*<0.001		0.78 (0.65, 0.93); *p*=0.004	
**Patients with ≥1 SABA canister prescription**				
n (%)	212 (6.6)	107 (3.3)	331 (29.2)	210 (18.5)
RR (95% CI); *p-value*†	0.50 (0.40, 0.64); *p*<0.001		0.63 (0.53, 0.75); *p*<0.001	

†Conditional Poisson regression; ‡the number of exacerbations may be counted multiple times per patient.

CI, confidence interval; MDV, Medical Data Vision; OCS, oral corticosteroid; PPPY, per-person-per-year; RR, rate ratio; SABA; short-acting β_2-agonist.

Supplementary Figure S1: Patient disposition.

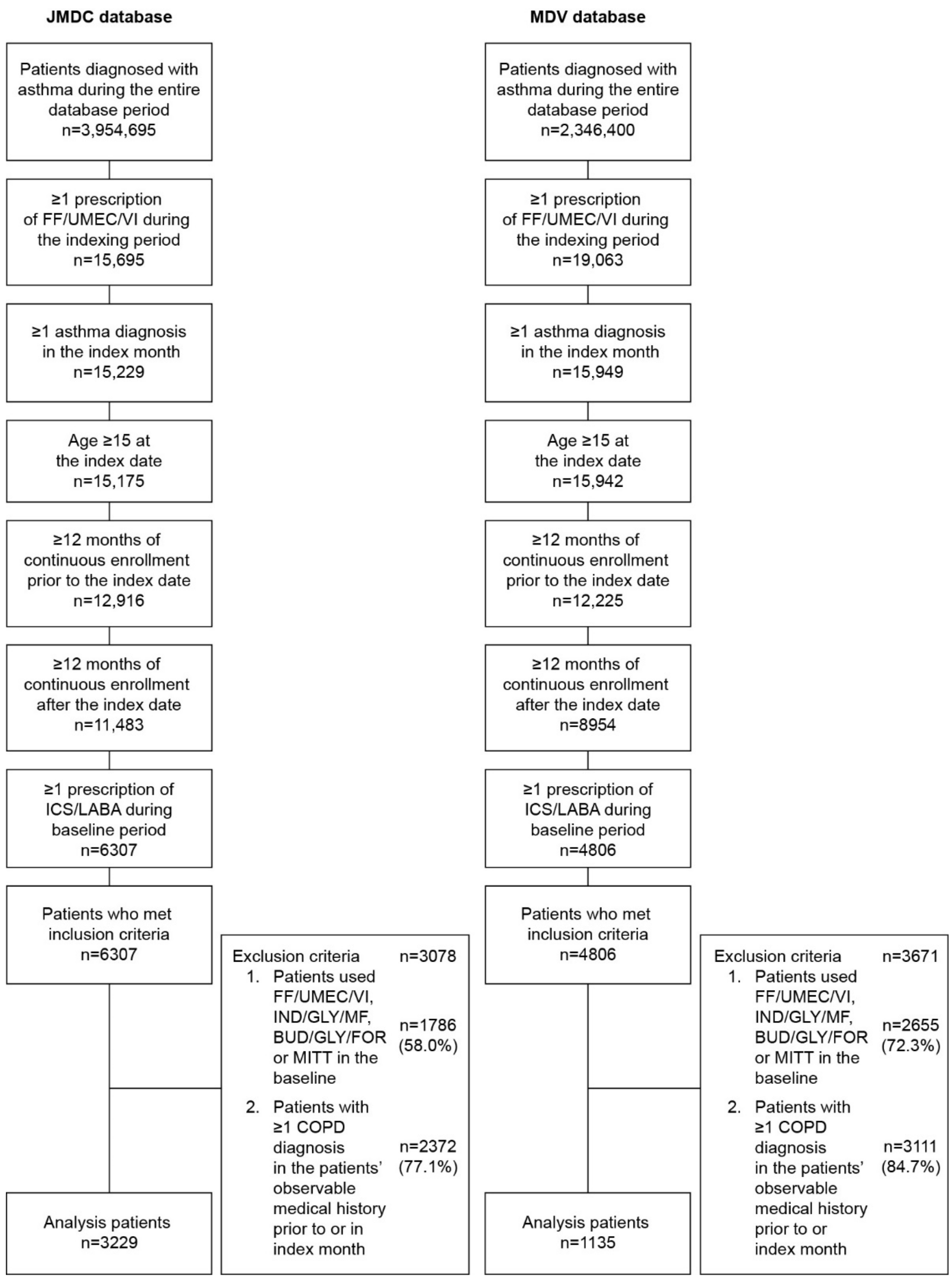

BUD, budesonide; COPD, chronic obstructive pulmonary disease; FF, fluticasone furoate; FOR, formoterol; GLY, glycopyrronium; ICS, inhaled corticosteroid; IND, indacaterol; LABA; long-acting β_2-agonist MDV, Medical Data Vision; MF, mometasone furoate; MITT, multiple-inhaler triple therapy; UMEC, umeclidinium; VI, vilanterol.
